# The Influenzal Pandemic

**Published:** 1918-11

**Authors:** 


### ABSTRACTS

### ACUTE RESPIRATORY INFECTIONS

***The Influenzal Pandemic.*** *British Medical Journal,* July 13, 1918, Editorial.

The onset is often peculiarly sudden, the victim being struck down with dizziness, weakness and pains in various parts of the body. There is a sharp rise in temperature to 103° or 104° F., with headache, pain in the back, and photophobia. The throat feels a little sore, pharynx is congested, and in some cases laryngitis and signs of bronchitis appear. Lateral nystagmus with suffusion of the conjunctivae, such as in trench or louse fever, has not rarely been found in these influenza patients. Few cases have proved fatal; in some, the cause of death has been acute bronchiolitis with increasing cyanosis and terminal failure of the right heart. Influenza of the gastro-intestinal type has not been common. Such a diagnosis, indeed, is held by many to be questionable and suspect at any time; and particularly so at the present moment when cases of food poisoning or acute disturbances, due to the unusual character of meals now-a-days, are not infrequently encountered. This circumstance has been cited to show that we are not now dealing with an epidemic or a pandemic of influenza at all; but bacteriological evidence is accumulating to prove that the influenza bacillus is responsible for at least a considerable proportion of the cases.

As for treatment, bed and the administration of salt of quinine or of aspirin seem to be indicated; the use of gargles appears not to influence the course of the disease.

In the matter of prophylaxis, as in the prophylaxis of cerebrospinal fever, free ventilation is imperative.

Without doubt, the virus of influenza is transmitted from one person to another in the vehicle of nasal and bucco-pharyngeal mucous, disseminated broadcast in the unguarded spasms of coughing, sneezing, and spitting, to which people with coughs and colds, whether influenzal or not, are objectionably prone.

